# The hospital burden of disease associated with bone metastases and skeletal-related events in patients with breast cancer, lung cancer, or prostate cancer in Spain

**DOI:** 10.1111/j.1365-2354.2009.01135.x

**Published:** 2010-11

**Authors:** RD Pockett, D Castellano, P McEwan, A Oglesby, BL Barber, K Chung

**Affiliations:** Cardiff Research Consortium, the MediCentreCardiff, UK; Hospital Universitario 12 de Octubre, Servicio de Oncologia MedicaCarretera Andalucia, Madrid, Spain; Cardiff Research Consortium, the MediCentreCardiff, UK; Global Health Economics, Amgen Inc.Thousand Oaks, CA, USA; Global Health Economics, Amgen Inc.Thousand Oaks, CA, USA; Global Health Economics, Amgen Inc.Thousand Oaks, CA, USA

**Keywords:** breast cancer, lung cancer, prostate cancer, metastases, skeletal-related events, burden of disease

## Abstract

POCKETT R.D., CASTELLANO D., MCEWAN P., OGLESBY A., BARBER B.L. & CHUNG K. (2010) *European Journal of Cancer Care***19**, 755–760 **The hospital burden of disease associated with bone metastases and skeletal-related events in patients with breast cancer, lung cancer, or prostate cancer in Spain**

Metastatic bone disease (MBD) is the most common cause of cancer pain and of serious skeletal-related events (SREs) reducing quality of life. Management of MBD involves a multimodal approach aimed at delaying the first SRE and reducing subsequent SREs. The objective of the study was to characterise the hospital burden of disease associated with MBD and SREs following breast, lung and prostate cancer in Spain. Patients admitted into a participating hospital, between 1 January 2003 and 31 December 2003, with one of the required cancers were identified and selected for inclusion into the study. The index admission to hospital, incidence of patients admitted and hospital length of stay were analysed. There were 28 162 patients identified with breast, lung and prostate cancer. The 3 year incidence rates of hospital admission due to MBD were 95 per 1000 for breast cancer, 156 per 1000 for lung cancer and 163 per 1000 for prostate cancer. For patients admitted following an SRE, the incidence rates were 211 per 1000 for breast cancer, 260 per 1000 for lung cancer and 150 per 1000 for prostate cancer. This study has shown that cancer patients consume progressively more hospital resources as MBD and subsequent SREs develop.

## INTRODUCTION

In the year 2002, there were approximately 162 000 new cases of cancer diagnosed in Spain: approximately 97 800 in males and 64 000 in females (ratio 1.5:1) (Ferlay *et al.* 2004). The overall incidence for Spain can be considered average for males and females with worldwide adjusted rates of 307 and 179 new cases respectively per 100 000 inhabitants per year. This ranks behind North America, Western Europe and Australia (López *et al.* 2005).

Overall, the most frequent cancer in Spain is colorectal (approximately 26 000 new cases per year), followed by lung (19 000), breast (16 000), bladder (14 500) and prostate (13 500). Breast cancer is the most common cancer in females, whereas in males the most common cancers are lung and prostate cancer.

Within the European context, the cancer survival rate in Spain is comparable with that of more developed countries and above the European average, regardless of cancer type (Gatta & the EUROCARE Working Group 2003). Overall, 44.0% of men, 56.4% of women, 76.4% of adolescents (14–18 years) and 71.0% of children (0–14 years) suffering from cancer in Spain, survived more than 5 years. The cancers with the worst prognosis are lung, oesophagus, pancreas and liver, with a 5-year survival rate at less than 20%, while breast, thyroid, bladder, melanoma, Hodgkin's lymphoma, uterine and testicular have the best prognosis with a 5-year survival at greater than 70%, and prostate cancer around 65% (Berrino & the EUROCARE Working Group 2003). Survival has improved by about 10% between the mid-1980s and the 1990s, and is expected to continue improving for most cancers (National Center for Epidemiology 2002).

Cancer is the leading cause of death among men and second among women, after cardiovascular diseases. In 2005, 96 499 people died in Spain from cancer, 60 701 males and 35 798 females (ratio 1.7:1) representing 25% of all deaths. Approximately one in three men (30.3%) and one in every five women (19.5%) die from cancer (López *et al.* 2004).

## METASTATIC BONE DISEASE AND SKELETAL-RELATED EVENTS

Bone metastases are a frequent complication of cancer, occurring in up to 70% of patients with advanced breast or prostate cancer and in approximately 15–30% of patients with lung, colon, stomach, bladder, uterus, rectum, thyroid, or kidney cancer (Coleman & Rubens 1987; Mundy 2002). Melanomas are other common cancers that metastasise to bone. Furthermore, once cancer metastasises to bone, it is usually incurable: only 20% of patients with breast cancer are still alive 5 years after the discovery of bone metastasis (Coleman 2001; Coleman & Seaman 2001).

Bone metastases are complicated by significant morbidity including skeletal-related events which are local irreversible changes and include pathological fracture, bone surgery, radiation therapy to the bone and spinal cord compression (Lorusso 2001; Coleman 2004; Lipton 2005; Delea *et al.* 2006). These events are widely considered to negatively affect quality of life (Lipton 2005; Wardley *et al.* 2005; Clemons *et al.* 2006), and hence present a challenge for the goals of palliative therapy, which include managing these patients' pain, preventing further deterioration and preserving quality of life (McMillan 1996; Ito & Tokudome 2006; Manders *et al.* 2006; Karamouzis *et al.* 2007).

Although the humanistic burden of skeletal-related events has been studied, little research has focused on the economic impact of these events, particularly in Europe. In the Netherlands, Groot *et al.* (2003) conducted hospital chart reviews of 28 patients with prostate cancer and metastatic bone disease (MBD) to quantify the cost associated with skeletal-related events. Costs and hospital length of stays varied by type of skeletal-related event and ranged from €1187 to €40 948, depending on event type.

The objective of the study was to characterise the hospital burden of disease associated with bone metastases and skeletal-related events following breast cancer, lung cancer and prostate cancer in Spain.

## METHODS

**Figure 1:**
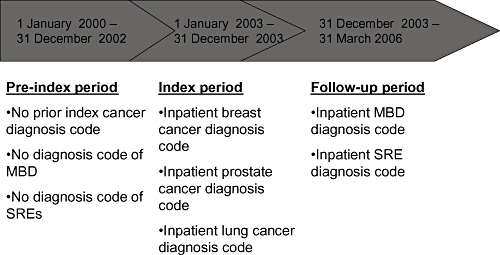
Study schema. MBD, metastatic bone disease; SRE, skeletal-related event.

Data between 1 January 2000 and 31 March 2006 were selected from the IASIST Conjunto Mínimo Básico de Datos (CMBD – the Joint Basic Minimum Dataset) hospital activity database which collects inpatient episode data for Spain. The database collects data on every hospital admission at 187 of 268 public (*n* = 156) and private hospitals (*n* = 31) across Spain. Patients with an index inpatient admission identifying either female breast cancer (ICD-9-CM 174*), lung cancer (ICD-9-CM 162*), or prostate cancer (ICD-9-CM 185*) between the dates of 1 January 2003 and 31 December 2003 were selected from CMBD. These patients were then traced back for 3 years (1 January 2000 to 31 December 2002), to identify incident cancer cases by excluding any patients who had previously been admitted with the same cancer. The remaining patients were then followed forward until 31 March 2006 to identify those with MBD (ICD-9-CM 198.5*), and subsequent skeletal-related events, specifically relating to pathological fractures, spinal cord compressions, radiation therapy, or bone surgery (Fig. 1), these were identified using ICD-9-CM codes for diseases, and for procedures. Patients who had an admission with bone metastases prior to a cancer admission, or an admission with a skeletal-related event prior to either an admission with cancer, or bone metastases, and patients who had an admission with a skeletal-related event but no admission with bone metastases were excluded from the study. The date of the index admission with cancer, bone metastases or skeletal-related event was not necessarily the date of diagnosis; however, for the purposes of this study it has been used as a proxy for date of diagnosis.

Descriptive statistical analysis was conducted for demographics within each cancer type to identify the number of patients in each category, mean age and sex, incidence rate for developing bone metastases following initial admission for cancer, and incidence rate for skeletal-related events following admission for bone metastases. Furthermore, the patient's hospital length of stay by index admission and subsequent re-admission, was analysed and described. Kaplan–Meier estimates were determined with regard to inpatient admissions for (1) time from index cancer to subsequent bone metastases; (2) time from bone metastases to first skeletal-related event; (3) time from first skeletal-related event to second skeletal-related event; and (4) time from second skeletal-related event to third skeletal-related event. Mortalilty data were not available for this dataset therefore only the time between admissions, and incidence of disease development has been included as true survival could not be calculated.

Finally, the cost for the index admission, and the cost of the subsequent admissions was analysed by specific disease stage and by cancer type. Each hospital has an associated cost negotiated annually with central government, while each medical procedure has a diagnosis-related group relative weight. The procedure cost was determined by multiplying the hospital cost by the diagnosis-related group relative weight. The cost applies to the admission and not necessarily to the treatment of the disease, therefore, the cost for the index admission is calculated using the admission where the cancer is first recorded, this was also the same for bone metastases and skeletal-related events.

## RESULTS

Between 1 January 2003 and 31 December 2003, there were 1 550 557 unique patients in the IASIST CMBD database, with 46 444 patients having a cancer admission and 28 162 with an index inpatient admission for either breast cancer (*n* = 10 090), lung cancer (*n* = 10 526), or prostate cancer (*n* = 7546) (Table 1). In these patients, the 3 year incidence rate of hospital admission due to subsequent bone metastases ranged from 95 per 1000 [95% confidence interval (CI) 89–101] for breast cancer, 156 per 1000 (95% CI 149–164) for lung cancer, males 153 per 1000 (95% CI 145–161), and females 179 per 1000 (95% CI 157–203), and 163 per 1000 (95% CI 155–172) for prostate cancer. Furthermore, the incidence rates for patients developing a skeletal-related event, subsequent to MBD, were 211 per 1000 (95% CI 183–242) for breast cancer, 260 per 1000 (95% CI 236–285) for lung cancer (males 260 per 1000 (95% CI 234–288), and females 258 per 1000 (95% CI 198–332), and 150 per 1000 (95% CI 131–171) for prostate cancer.

**Table 1:** Annual number of patients presenting at secondary care with first recorded cancer, bone metastases, or skeletal-related event (SRE) clinical code

	Cancer only	Cancer with bone metastases only	Cancer with bone metastases and SRE
Breast cancer			
Number	9136	753	201
Mean age	60	62	61
Female	100%	100%	100%
Lung cancer			
Number	8881	1218	427
Mean age	67	64	62
Male	87.8%	85.6%	85.7%
Female	12.2%	14.4%	14.3%
Prostate cancer			
Number	6293	1031	221
Mean age	73	74	73
Male	100%	100%	100%

### Length of stay

**Figure 2:**
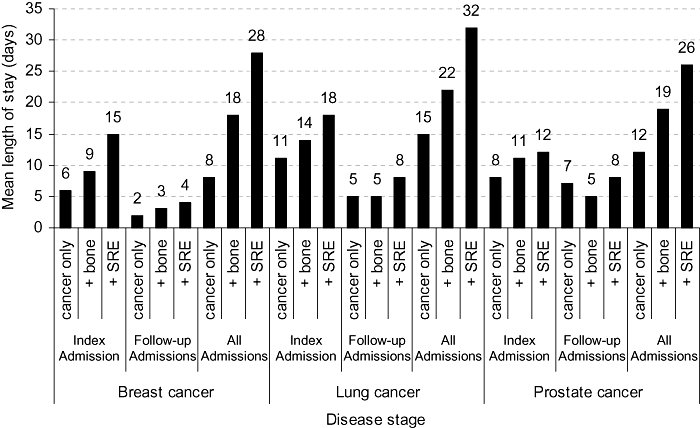
Mean length of stay by disease type and stage. SRE, skeletal-related event.

Patient length of hospital stay increased with the development of bone metastases and skeletal-related events (Fig. 2); this observation was consistent across cancer types. The length of stay for the index admission was greatest in those who subsequently developed skeletal-related events (12–18 days), compared with those with the cancer only (6–11 days) and cancer and MBD (9–11 days); furthermore, follow-up attendances also had greater length of stays in patients who subsequently developed skeletal-related events (4–8 days), again compared with those with the cancer only (2–7 days). Similarly, the length of stay over 3 years across all related admissions showed the same pattern with those developing skeletal-related events having a far greater overall mean length of stay (26–32 days) than those who developed no further complications (8–15 days), or who developed MBD (18–22 days). Of those with skeletal-related events, the hospital length of stay varied between event type with index admissions averaging between 12 and 20 days, follow-up admissions between 3 and 11 days, and overall length of stay between 26 and 35 days (Table 2).

**Table 2:** Length of stay by skeletal-related event type

	Index Admission	Follow-up Admission	All Admissions
Breast cancer			
Pathological fracture	16	5	31
Spinal cord compression	14	9	27
Bone surgery	17	7	35
Radiation therapy	16	3	30
Lung cancer			
Pathological fracture	20	7	32
Spinal cord compression	15	7	28
Bone surgery	19	11	35
Radiation therapy	19	10	34
Prostate cancer			
Pathological fracture	12	10	26
Spinal cord compression	14	7	27
Bone surgery	12	11	29
Radiation therapy	13	7	27

**Figure 3:**
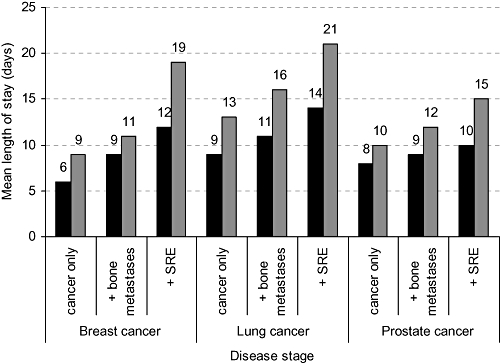
Mean length of stay by disease type and admission status. SRE, skeletal-related event. 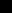 elective, 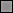 emergency.

Stratifying the data by admission type (elective or emergency) demonstrated the increase in 3-year length of stay statistics attributable to bone metastases and skeletal-related events was largely driven by patients with emergency admissions (Fig. 3).

### Re-admissions and survival

Patients with MBD and skeletal-related events generally had a greater mean number of re-admissions to hospital than those with cancer only. However, there was no difference in the number of readmissions between those who developed bone metastases only and those who developed MBD followed by a skeletal-related event. The average number of re-admissions was one in patients with breast, lung and prostate cancer only, while in those with MBD the average number or re-admissions was three, one and two, for breast, lung or prostate cancer, respectively.

### Cost

**Figure 4:**
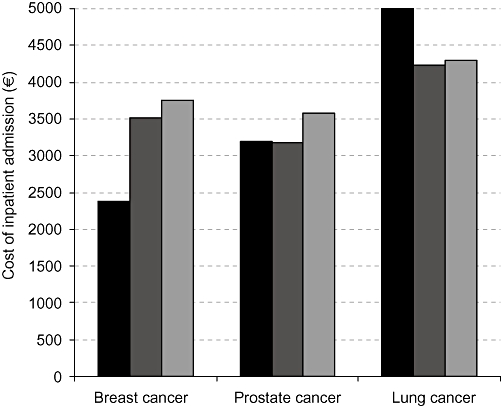
Inpatient costs by first (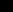) index cancer admission, first (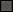) MBD admission, and first (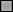) SRE admission. MBD, metastatic bone disease; SRE, skeletal-related event.

The pattern of costs was similar across breast and prostate cancer types, showing that the cost of the admissions increased as the disease progressed into MBD and subsequent skeletal-related events (Fig. 4). The average cost of the index admission for those with breast cancer was €2374 (SD €1216.58); with the average cost of the first admission with MBD increasing to €3515 (SD €1530.99); and €3757 (SD €1420.89) for the first admission with a skeletal-related event, while for prostate cancer patients the average costs were €3194 (SD €2509.68); €3180 (SD €2081.85) and €3585 (SD €1538.82). In contrast, the index admission cost for lung cancer patients was higher than subsequent admissions for MBD or skeletal-related events [€4994 (SD €3765.16), €4227 (SD €2037.45) and €4298 (SD €1939.85), respectively].

## DISCUSSION

It was shown that within 3 years of their index admission 9.5% of breast cancer patients, 15.6% of lung cancer patients and 16.6% of prostate cancer patients were subsequently admitted to secondary care for development of bone metastases. Furthermore, 21.1% of these breast cancer patients, 26.0% of these lung cancer patients and 17.7% of these prostate cancer patients later developed a skeletal-related event requiring hospital admission.

As MBD and skeletal-related events are signs of progression in cancer patients, our findings, which indicate that patients who develop MBD or subsequent skeletal-related events are more health resource intensive when compared with patients who have cancer only, is not unexpected. Not only are their inpatient lengths of stay longer, through their index and follow-up admissions, they are also re-admitted more often. In addition, once a patient has developed bone metastases and a skeletal-related event, the risk for subsequent events increases with the time between re-admissions for skeletal-related events becoming shorter.

The associated cost of the index admission was generally increased for people who subsequently developed MBD when compared with those who did not; however, this cost increased further once the bone metastases had developed. This was also observed in patients who developed skeletal-related events with the cost of treatment increasing further again. This could be indicative of patients who later develop MBD and skeletal-related events having more advanced cancer at the index admission, with advancing cancer likely to lead to a longer length of stay and a subsequent reduction in the quality of life (Coleman 2001; Wardley *et al.* 2005; Weinfurt *et al.* 2005; DePuy *et al.* 2007), therefore requiring more care, and the requirement for more complicated, and expensive, medical and surgical procedures.

It has been shown previously that as cancer progresses and becomes more advanced, quality of life decreases, prognosis worsens and the cost of treatment increases (Groot *et al.* 2003). This study confirms that patients who develop MBD subsequent to their index hospital admission for cancer are a greater burden to health service providers than those who have cancer only, and this burden further increases in those who subsequently further develop a skeletal-related event. By reducing the number of patients with cancer from developing bone metastases, and furthermore reducing the number who develop skeletal-related events both the financial and logistical burden on health service providers could be greatly reduced.

## LIMITATIONS

As the study was undertaken using secondary care data, it is prudent to be aware that limitations exist. As with all databases such as IASIST's CMBD, there is the potential for miscoding to take place and therefore patients may have been included or excluded when they should not have been. Furthermore, patients have been included in the study because of the presence of the specific disease being recorded at that hospital attendance whether the attendance was related to that disease or not, it is therefore also likely that the true date of diagnosis is earlier than that recorded in the inpatient records, which has been used as a proxy. Patient mortality is not included in CMBD, therefore, patient re-admissions and survival to further admissions may be skewed towards a better prognosis.

Cancer care may have been provided in other settings, such as by primary care providers, outpatient attendances, or in hospitals not providing data to CMBD, therefore, it is possible that the true incidence and costs of cancer is being underestimated.

## CONCLUSIONS

This study has shown that cancer patients consume progressively more hospital resources when they develop bone metastases and subsequent skeletal-related events. Reducing the incidence of MBD and skeletal-related events may lead to less inpatient admissions, shorter lengths of stay and smaller costs.
